# Establishment of a novel cytokine-related 8-gene signature for distinguishing and predicting the prognosis of triple-negative breast cancer

**DOI:** 10.3389/fmed.2023.1189361

**Published:** 2023-06-02

**Authors:** Xiaojun Liu, Liang Zhang, Liang Chen

**Affiliations:** Second Department of Nail and Breast Surgery, Cangzhou Central Hospital, Cangzhou, China

**Keywords:** triple-negative breast cancer, prognosis, cytokine-related pathway, cytokine-related signature, biomarkers

## Abstract

**Background:**

Triple-negative breast cancer (TNBC) is a common carcinoma in women, and the prognosis of TNBC is the worst. Using data from The Cancer Genome Atlas (TCGA) database, we analyzed the functional roles of cytokine-related genes in TNBC.

**Methods:**

The clinical and transcriptome data of TNBC patients were downloaded from TCGA database. A systematical analyses of the data from TCGA database were conducted to screen the prognostic genes and identify the main cytokine-related pathways related to TNBC.

**Results:**

We identified 499 prognostic genes in TNBC patients from TCGA database and the cytokine-related pathways closely related to TNBC. TCGA-TNBC patients were divided into the high-risk cluster (C1) group and the low-risk cluster (C2) group based on the cytokine-related genes. The C1 group patients exhibited tumor metastasis and an advanced tumor stage. The functional analysis revealed that the upregulated differentially expressed genes (DEGs) in the C1 group were mainly associated with the extracellular matrix (ECM)-receptor interaction, stem cell proliferation, focal adhesion, and cyclic adenosine monophosphate (cAMP) signaling pathway, while the downregulated DEGs in the C1 group were mainly associated with cytokine and cytokine receptors, T-helper 17 (Th17) cell differentiation, and primary immunodeficiency. The immune activity of C1 group was lower than that of C2 group, and the identified half-maximal inhibitory concentration scores of 3 chemotherapy drugs (i.e., doxorubicin, methotrexate, and paclitaxel) were lower in C2 group than C1 group. More importantly, we constructed a novel prognostic signature and identified the following 8 genes: CCL25, CXCL13, IL12RB2, IL21, TNFRSF13C, TNFRSF8, CCL7 and GDF5.

**Conclusion:**

The status of the cytokine-related pathway was closely related to tumor classification and immune activity in the TNBC patients. The gene signature of the cytokine-related genes showed an good performance in predicting the prognosis of TNBC patients, and could predict the prognosis of TNBC patients.

## Introduction

Breast cancer (BC) is a global health issue and one of the principal causes of female morbidity according to the World Health Organization (1). Triple-negative breast cancer (TNBC) accounts for 10–15% of BCs, and has the worst prognosis of all BCs (2). TNBC is characterized by lack of estrogen receptor expression, progesterone receptor expression, human epidermal growth factor receptor 2 overexpression or gene amplification (3). Like other clinical subtypes of BC, TNBC is biologically heterogeneous (4), which leads to the diversification of clinical and epidemiological behaviors.

Currently, there is no tumor-specific targeted therapy for TNBC. Due to the optimization of chemotherapy regimens and the emergence of immunotherapy, the clinical outcomes of early stage TNBC patients have improved (5). However, research still needs to be conducted to develop targeted drug treatments that can target mutations and prevent immune escape, and socioeconomic support needs to be increased (3–5). At the same time, TNBC patients currently lacks specific and effective targeted therapy.

Cytokines, which are secreted by various cell types and are regulators of cell activity, are key proteins in signal transduction in the tumor microenvironment (TME) and have pleiotropic effects (6). Cytokines can be divided into interleukins (ILs), interferons, tumor necrosis factor (TNF) superfamily, colony-stimulating factors, chemokines, growth factors, etc. (7). IL signaling in cancer cells is associated with tumor growth and metastatic mechanisms (8). IL-1 not only promotes inflammation-induced carcinogenesis, but also contributes to tumor invasion and angiogenesis (9). IL-18 promotes angiogenesis, induces cancer cell proliferation and invasion, and prevents cell apoptosis by activating nuclear factor kappa B (10). IL-6 also promotes carcinogenesis in chronic inflammation and drives tumor-intrinsic progression (11). Additionally, high-dose recombinant IL-2 has been approved by the Food Drug Administration (FDA) for the treatment of metastatic renal cell carcinoma (RCC) and melanoma (12). Recombinant human IL-7 or a modified form of IL-7 fused to immunoglobulin G fragment crystallizable fragments can effectively induce an inflammatory TME (13). In BC, especially TNBC, the number of patients who will benefit from immune-checkpoint inhibitor (ICI) treatment is small (14, 15), and it has been suggested that the efficacy of therapy in BC patients is limited. Thus, it is of great importance to identify novel biomarkers and clarify the relationship between the cytokine-related pathway and prognosis in TNBC.

In this study, we summarized the molecular typing of TNBC based on cytokine-related gene signatures and constructed risk-prediction models. Our findings provided new perspectives on the early detection, prognosis, and treatment optimization of TNBC.

## Methods

### Data sets of TNBC patients

The raw data and clinical details of 160 TNBC patients were downloaded from The Cancer Genome Atlas (TCGA) database.^1^

### Identification of prognostic genes

We first conducted a univariate Cox analysis to explore the roles of prognostic genes in TNBC patients and displayed the top 20 prognostic genes according to their *p* values. Functional annotation analyses [i.e., a Kyoto Encyclopedia of Genes and Genomes (KEGG) enrichment analyses and a Gene Ontology (GO) analysis] of the prognostic genes were also conducted using the “ClusterProfiler” R package. The results were visualized using the “ggplot2” R package. Comprehensive protein–protein interaction (PPI) networks were constructed using R cluster Profilter.

### Tumor classification based on the cytokine-related pathway in TNBC

The consensus clustering analysis was conducted using the R package ConsensusClusterPlus (version 1.54.0), and the clustering variable (kappa) was set from 2–6. The heatmap analysis was performed using the pheatmap R package (version 1.0.12). The Kaplan–Meier curves were plotted using the “survival” R package.

### Functional annotation in 2 clusters of TNBC

The differentially expressed genes (DEGs) between cluster 1 (C1) and cluster 2 (C2) were identified based on a |log 2 fold change| >1 and *p* value <0.05 using the “DESeq2” R package. Volcano plots were generated to display the DEGs. The top 50 DEGs were shown in a heatmap generated by the “pheatmap” R package (version 1.0.12). The GO and KEGG analyses were performed using the “ClusterProfiler” R package.

### Comparison of immune activity in the 2 clusters of TNBC

Immune infiltration was estimated using CIBERSORT in “immunedeconv” and visualized in heatmaps and boxplots. The following 8 immune checkpoint-related genes were selected: *CD274*, *PDCD1*, *PDCD1LG2*, *CTLA4*, *LAG3*, *HAVCR2*, *TIGIT*, and *SIGLEC15*. The gene expression levels were determined and visualized by the “ggplot2” and “pheatmap” R packages. The statistical analyses were performed using the Wilcox test, and a *p* value <0.05 was considered statistically significant.

### Drug sensitivity analysis of the two clusters

Drug susceptibility was assessed in the 2 clusters using the pRRophetic algorithm. The chemotherapeutic response for each sample was predicted based on the available pharmacogenomics database.^2^ The prediction process was implemented by the“pRRophetic” R package. The half-maximal inhibitory concentration (IC50) was estimated by ridge regression, and a *p* value <0.05 was considered statistically significant.

### A prognostic gene model based on the cytokine-related genes

The prognostic value of the cytokine-related genes was analyzed. A least absolute shrinkage and selection operator (LASSO) Cox regression analysis was conducted to develop a prognostic model using the “glmnet”R package. The variables used to construct the prognostic model were non-zero coefficients and the lambda condition was the minimum. The risk score was calculated as follows: risk score = sum (expression level of each gene × corresponding coefficient). A Kaplan–Meier analysis was performed using the “survival” R package. A Cox proportional-hazards analysis was conducted to calculate the hazard ratios (HRs) with 95% confidence intervals (CIs). Moreover, the immunohistochemical expression of cytokine-related genes were validated between normal tissues and BC tissues from the HPA database.^3^

## Results

### Functional enrichment analyses of the prognostic genes in TNBC

**Figure 1:**
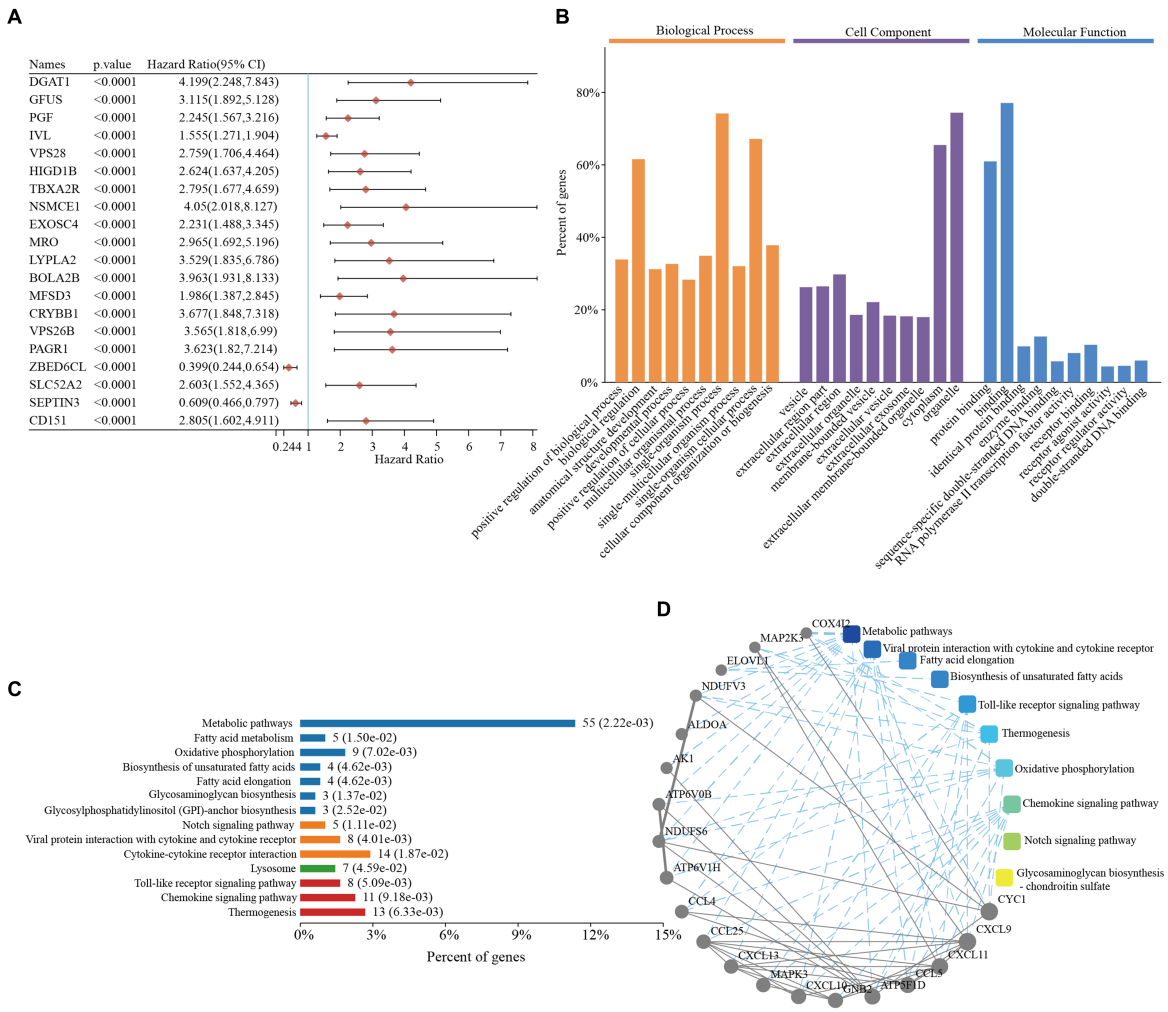
Functional enrichment analyses of prognostic genes in triple-negative breast cancer. **(A)** The top 20 prognostic genes in triple-negative breast cancer. **(B)** Gene Ontology enrichment analysis of the prognostic genes in triple-negative breast cancer. **(C)** The KEGG enrichment pathways of the prognostic genes in triple-negative breast cancer. **(D)** PPI networks of the prognostic genes in triple-negative breast cancer. CI, confidence interval; KEGG, Kyoto Encyclopedia of Genes and Genomes; PPI, protein–protein interaction.

We first explored the roles of the prognostic genes in the TNBC patients. In total, 499 prognostic genes were identified in TNBC, and the top 20 genes (as determined by the univariate Cox analysis) are presented in Figure 1A (Supplementary Table S1). Functional enrichment analyses of the 499 prognostic genes were then performed. The GO enrichment analysis revealed that these genes were mainly involved in biological regulation, receptor regulator activity, and receptor binding (Figure 1B and Supplementary Table S1). Similarly, the KEGG enrichment results revealed that these genes were mainly involved in the cytokine-cytokine receptors, chemokine signaling pathway, and metabolic pathways (Figure 1C and Supplementary Table S1), which suggested that these pathways might have important roles in TNBC. Comprehensive PPI networks were constructed and we also identified the chemokine-related pathway that was closely related to TNBC (Figure 1D and Supplementary Table S1).

### Tumor classification based on the cytokine-related pathway in TNBC

As cytokine- and chemokine-related pathways are closely related to TNBC, we focused on exploring their functional roles in TNBC. We investigated the expression of the 14 cytokine-related genes [i.e., chemokine ligand 25 (*CCL25*), chemokine CXC motif ligand 13 (*CXCL13*), IL-12 receptor subunit beta-2 (*IL12RB2*), Interferon gamma (*IFNG*), Interleukin-21 (*IL21*), chemokine CXC motif ligand 11 (*CXCL11*), transmembrane activator and calcium modulator and cyclophilin ligand interactor (*TNFRSF13C*), chemokine CXC motif ligand 10 (*CXCL10*), chemokine CXC motif ligand 9 (*CXCL9*), chemokine (CC motif) ligand 4 (*CCL4*), tumor necrosis factor receptor superfamily member 8 (*TNFRSF8*), chemokine (CC motif) ligand 5 (*CCL5*), chemokine (CC motif) ligand 7 (*CCL7*), and growth/differentiation factor-5 (*GDF5*)] involved in the TNBC subtypes. A consistency cluster analysis was conducted to divide TCGA-TNBC patients into different clusters based on these genes.

**Figure 2:**
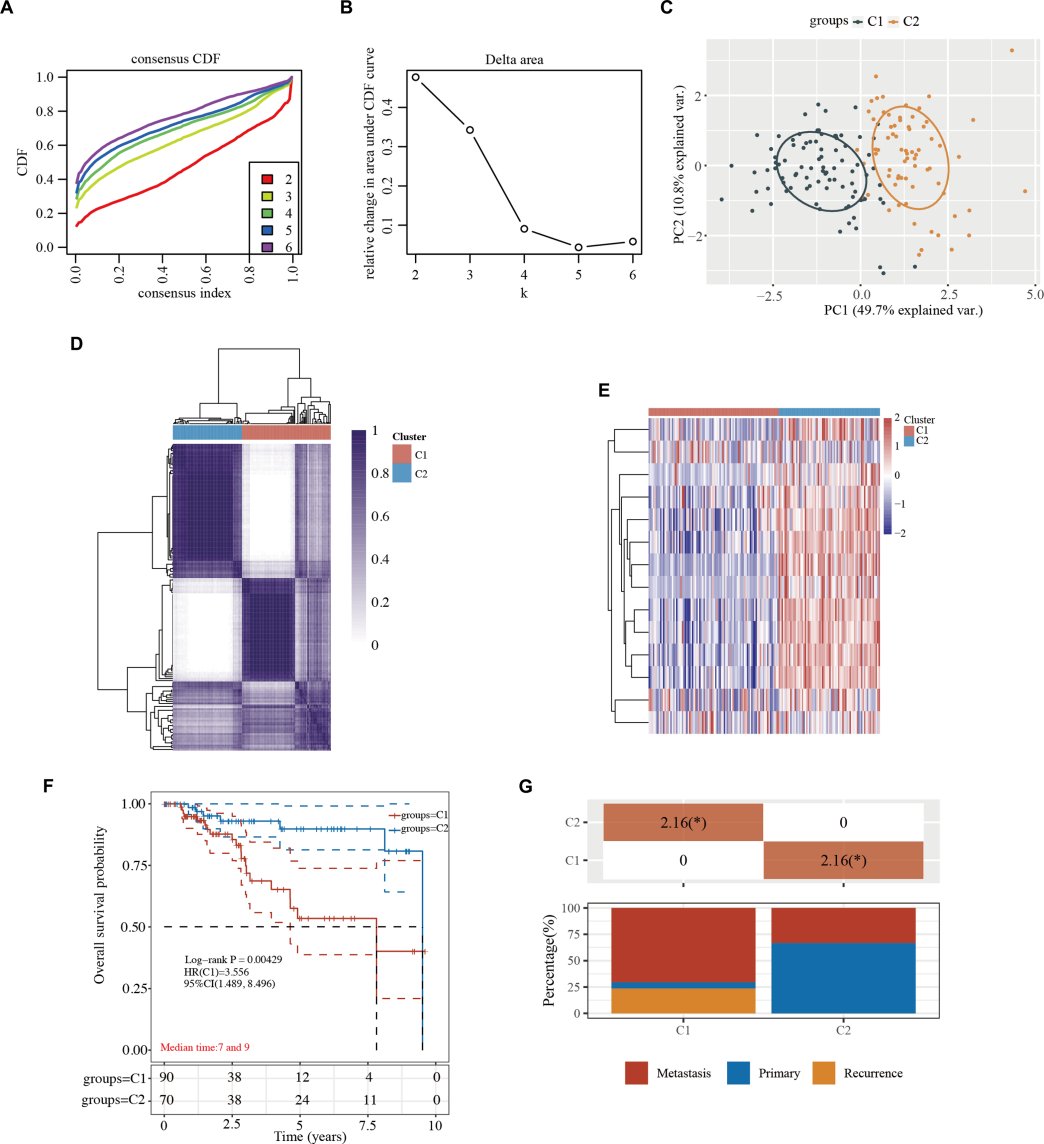
Tumor classification based on the cytokine-related pathway in TNBC. **(A)** CDF plot for k = 2 to 6. **(B)** AUC changes. **(C)** The principal component analysis of the 2 clusters. **(D)** Consensus clustering matrix of the 2 clusters in TNBC (k = 2). **(E)** Heatmap of the 2 clusters in TNBC. **(F)** Kaplan–Meier survival curves of overall survival in the 2 clusters. **(G)** Kaplan–Meier survival curves of overall survival in the 2 clusters. **(G)** Distribution of stage in the 2 clusters. ^*^, indicates a significant difference between the 2 clusters (*p* < 0.05). CDF, cumulative distribution function; HR, hazard ratio; CI, confidence interval; TNBC, triple-negative breast cancer; AUC, area under the curve.

When the clustering variable (kappa) was 2 (Figures 2A,B), TCGA-TNBC patients were divided into 2 clusters (i.e., C1 and C2) using a consistency cluster analysis and principal component analysis (PCA) (Figures 2C,D and Supplementary Table S2). The 2 clusters of TCGA-TNBC patients were well separated in the heatmap (Figure 2E and Supplementary Table S2). The overall survival (OS) times of TCGA-TNBC patients were compared between the 2 clusters. The Kaplan–Meier curves revealed that the TNBC patients in C1 had better survival than those in C2 (HR: 3.556, 95% CI: 1.489–8.496, *p* = 0.00429; Figure 2F and Supplementary Table S2). Furthermore, the TNBC C1 patients exhibited tumor metastasis and an advanced tumor stage (Figure 2G and Supplementary Table S2).

### Functional annotation in the 2 clusters of TNBC

**Figure 3:**
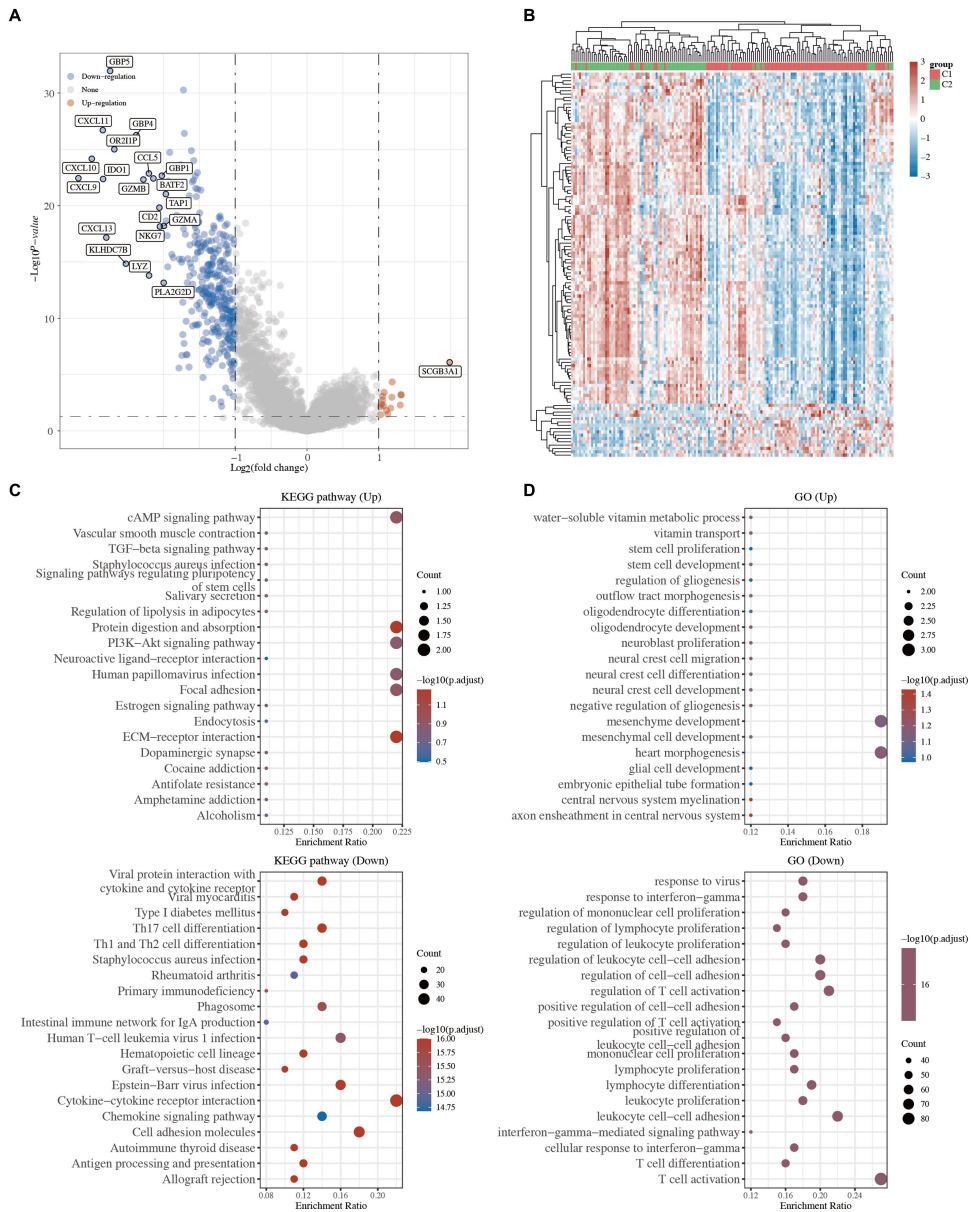
Functional annotation between the 2 clusters in TNBC. **(A)** Volcano plot of the DEGs. Red and blue represent the upregulated and downregulated genes, respectively. **(B)** Heatmap of the top 50 differentially expressed genes. **(C)** KEGG pathway analysis of the upregulated and downregulated DEGs. **(D)** GO analysis of the upregulated and downregulated DEGs. KEGG, Kyoto Encyclopedia of Genes and Genomes; GO, Gene Ontology; TGF, transforming growth factor; ECM, extracellular matrix; IgA, immunoglobulin A; TNBC, triple-negative breast cancer; DEGs, differentially expressed genes.

To investigate the underlying mechanism of these 2 TNBC clusters, 16 upregulated and 328 downregulated genes were identified in the volcano plot (C1 vs. C2; Figure 3A and Supplementary Table S3). The top 50 significant DEGs are presented in a heatmap, which showed the different characteristics of the 2 TNBC clusters (Figure 3B and Supplementary Table S3).

GO and KEGG enrichment analyses were carried out to compare the biological roles of these DEGs between C1 and C2. The KEGG analysis revealed that the pathways of the upregulated genes in C1 were mainly associated with the extracellular matrix (ECM)-receptor interaction, focal adhesion, and cyclic adenosine monophosphate (cAMP) signaling pathway, while the pathways of the downregulated genes in C1 were mainly associated with the viral protein interaction with cytokine and cytokine receptors, T-helper 17 (Th17) cell differentiation, and primary immunodeficiency (Figure 3C and Supplementary Table S3). Similarly, the GO analysis revealed that the upregulated genes were mainly related to stem cell proliferation, stem cell development, and embryonic epithelial tube formation, while the downregulated genes were mainly related to immune-related processes (Figure 3D and Supplementary Table S3). Several studies have shown that ECM-receptor interaction, focal adhesion, and stem cell proliferation are hallmarks of cancer (16–18). These results suggested that the tumor cells of the C1 TNBC patients might have a high proliferative ability.

### Comparison of immune activity in the 2 clusters of TNBC

**Figure 4:**
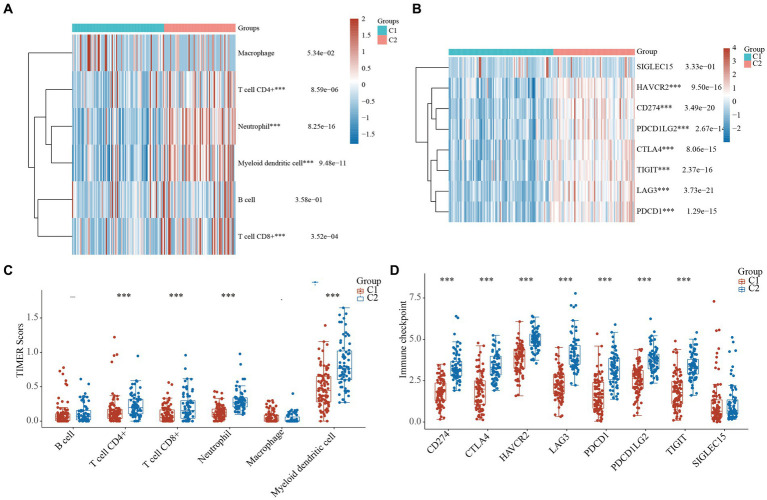
Comparison of immune activity in the 2 clusters of TNBC. **(A)** Heatmap of cytokine-related genes between the 2 clusters. **(B)** Boxplots of the cytokine-related genes between the 2 clusters. Heatmap **(C)** and boxplot **(D)** of the gene expression of the immune checkpoint inhibitors between the 2 clusters. ^***^, *p* < 0.001. TIMER, tumor immune estimation resource; TNBC, triple-negative breast cancer.

It has been reported that cytokines are closely linked to immune activity in many cancers (6–8). Thus, we compared the immune activity of the 2 clusters of TNBC patients. The heatmaps and boxplots showed that the immune cells, including the T cells, neutrophils, and myeloid dendritic cells, of the C1 TNBC patients differed significantly to those of the C2 TNBC patients (Figures 4A,B and Supplementary Table S4). Furthermore, the statuses of the ICIs were analyzed between the 2 clusters. The heatmap and boxplots showed that 7 of the 8 ICI-related genes (i.e., *LAG3*, *PDCD1*, *CTLA4*, *TIGIT*, *HAVCR2*, *CD274*, and *PDCD1LG2*) were lower in the C1 TNBC patients than the C2 TNBC patients (Figures 4C,D and Supplementary Table S4). These results indicated a close link between cytokines and immune activity.

### Drug sensitivity analysis between the 2 clusters

**Figure 5:**
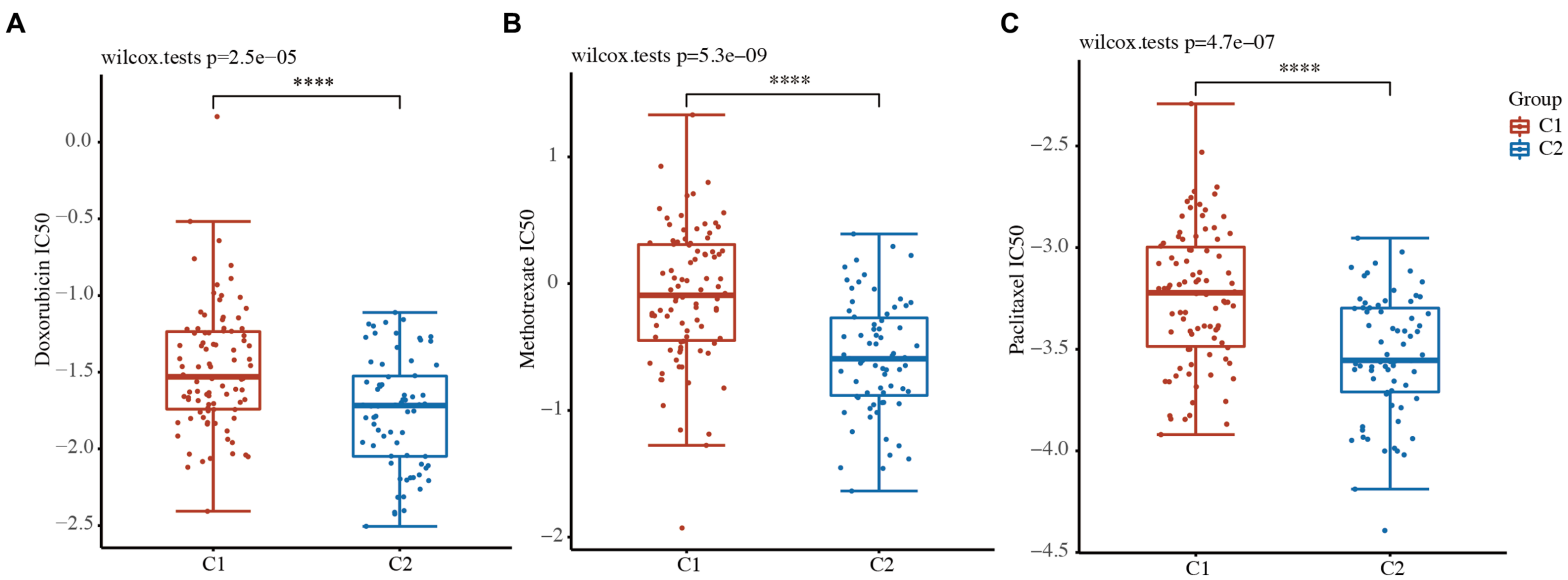
Drug sensitivity analysis between the 2 clusters. **(A-C)** IC50 values of doxorubicin **(A)**, methotrexate **(B)**, and paclitaxel **(C)** between the 2 clusters. ^****^, *p* < 0.0001.

Drug susceptibility was assessed in the 2 clusters based on the pRRophetic algorithm. The status of the 2 clusters was closely related to the IC50 scores of doxorubicin, methotrexate, and paclitaxel in the TNBC patients (Figures 5A–C and Supplementary Table S5). The results revealed that the IC50 values of doxorubicin, methotrexate, and paclitaxel were lower in the low-risk C2 TNBC patients than C1 TNBC patients (Figures 5A–C and Supplementary Table S5). These results confirmed that cytokine-related classification might be a good predictor of the successful use of chemotherapy.

### A prognostic gene model based on cytokine-related genes

**Figure 6:**
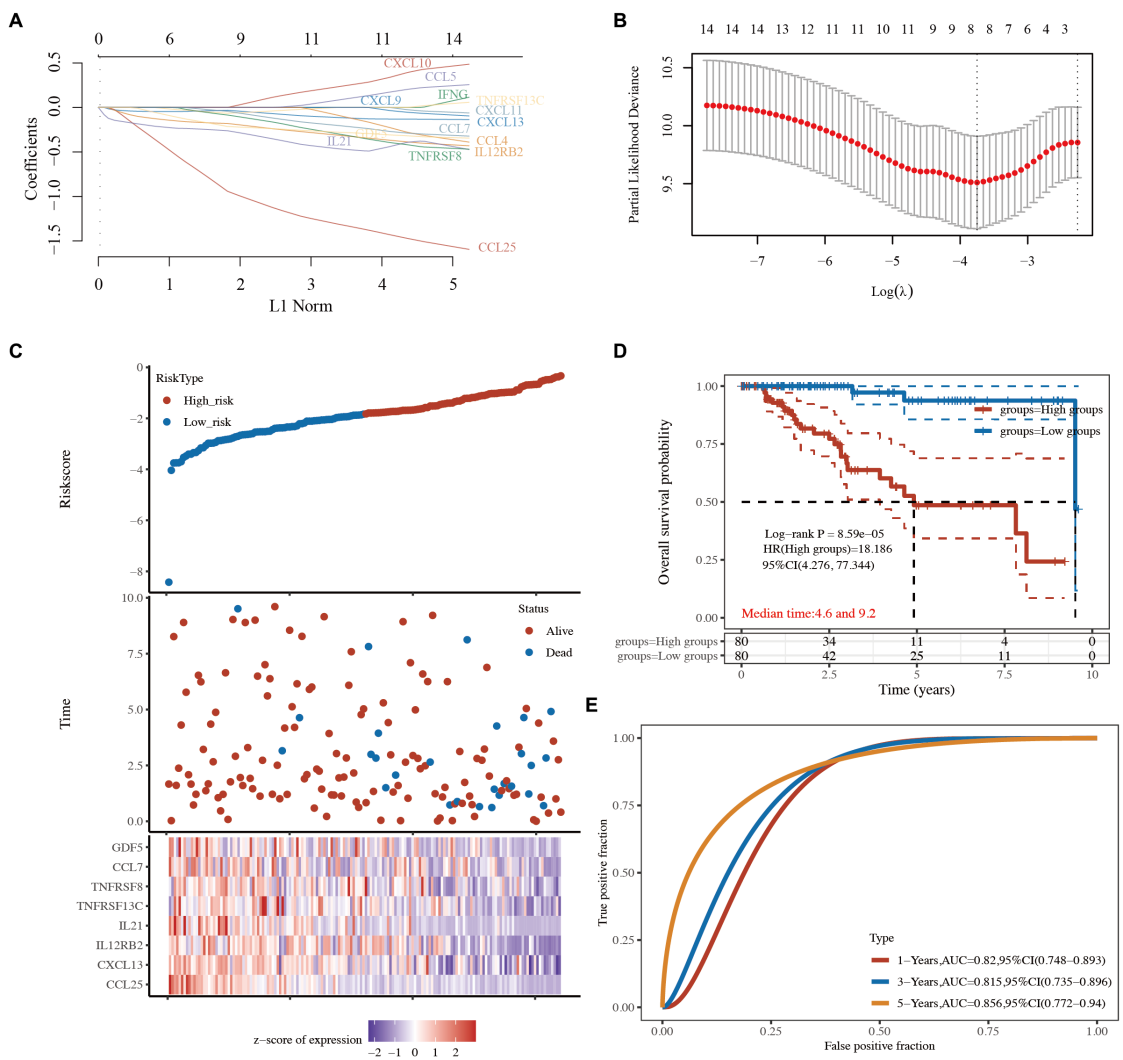
Prognostic predictive signatures of the cytokine-related genes in TCGA-TNBC cohort. **(A)** LASSO coefficient profiles of the 14 cytokine-related genes. **(B)** Partial likelihood deviance was plotted. **(C)** Risk score for each TNBC patient based on the cytokine-related genes. TCGA-TNBC patients were divided into the high-risk group and low-risk group based on the median risk score. The levels of the 8 prognostic genes in the high-risk group and low-risk group are shown in red and blue, respectively. **(D)** Kaplan–Meier survival curves of the 2 groups. **(E)** The AUC of the risk score signature. LASSO, least absolute shrinkage and selection operator; TCGA, The Cancer Genome Atlas; TNBC, triple-negative breast cancer; AUC, area under the curve.

To further confirm the prognostic value of the 14 cytokine-related genes, a prognostic model was constructed to optimize these cytokine-related genes using the LASSO regression algorithm. The following 8 cytokine-related genes related to TCGA-TNBC prognosis were identified: *CCL25*, *CXCL13*, *IL12RB2*, *IL21*, *TNFRSF13C*, *TNFRSF8*, *CCL7*, and *GDF5* (Figures 6A,B and Supplementary Table S6). The risk score was calculated as follows:


$$\begin{array}{l} \mathrm{Risk score}=\left(-0.814\times CCL25\;\mathrm{expression}\right)+\\ \;\;\;\;\;\;\;\;\;\;\;\;\;\;\;\;\;\;\;\;\;\;\;\;\;\;\;\;\;\;\;\left(-0.0363\times CXCL13\;\mathrm{expression}\right)+\\ \;\;\;\;\;\;\;\;\;\;\;\;\;\;\;\;\;\;\;\;\;\;\;\;\;\;\;\;\;\;\;\left(-0.1737\times IL12RB2\;\mathrm{expression}\right)+\\ \;\;\;\;\;\;\;\;\;\;\;\;\;\;\;\;\;\;\;\;\;\;\;\;\;\;\;\;\;\;\;\left(-0.2503\times IL21\;\mathrm{expression}\right)+\\ \;\;\;\;\;\;\;\;\;\;\;\;\;\;\;\;\;\;\;\;\;\;\;\;\;\;\;\;\;\;\;\left(-0.0532\times TNFRSF13C\;\mathrm{expression}\right)+\\ \;\;\;\;\;\;\;\;\;\;\;\;\;\;\;\;\;\;\;\;\;\;\;\;\;\;\;\;\;\;\;\left(-0.063\times TNFRSF8\;\mathrm{expression}\right)+\\ \;\;\;\;\;\;\;\;\;\;\;\;\;\;\;\;\;\;\;\;\;\;\;\;\;\;\;\;\;\;\;\left(-0.028\times CCL7\;\mathrm{expression}\right)+\\ \;\;\;\;\;\;\;\;\;\;\;\;\;\;\;\;\;\;\;\;\;\;\;\;\;\;\;\;\;\;\;\left(-0.1811\times GDF5\;\mathrm{expression}\right) \end{array}$$

TCGA-TNBC patients were divided into the high-risk group and low-risk group based on the median risk score (Figure 6C). The Kaplan–Meier curves showed that the low-risk group had better survival than the high-risk group (HR: 18.186, 95% CI: 4.276–77.344, *p* = 8.59e-05; Figure 6D and Supplementary Table S6). A receiver operating characteristic (ROC) curve was used to evaluate the prognostic performance of the 8 cytokine-related genes. The areas under the ROC curves (AUCs) were 0.820, 0.815, and 0.856 for 1-, 3-, and 5-year OS, respectively (Figure 6E and Supplementary Table S6), indicating that the model had a good prognostic effect.

**Figure 7:**
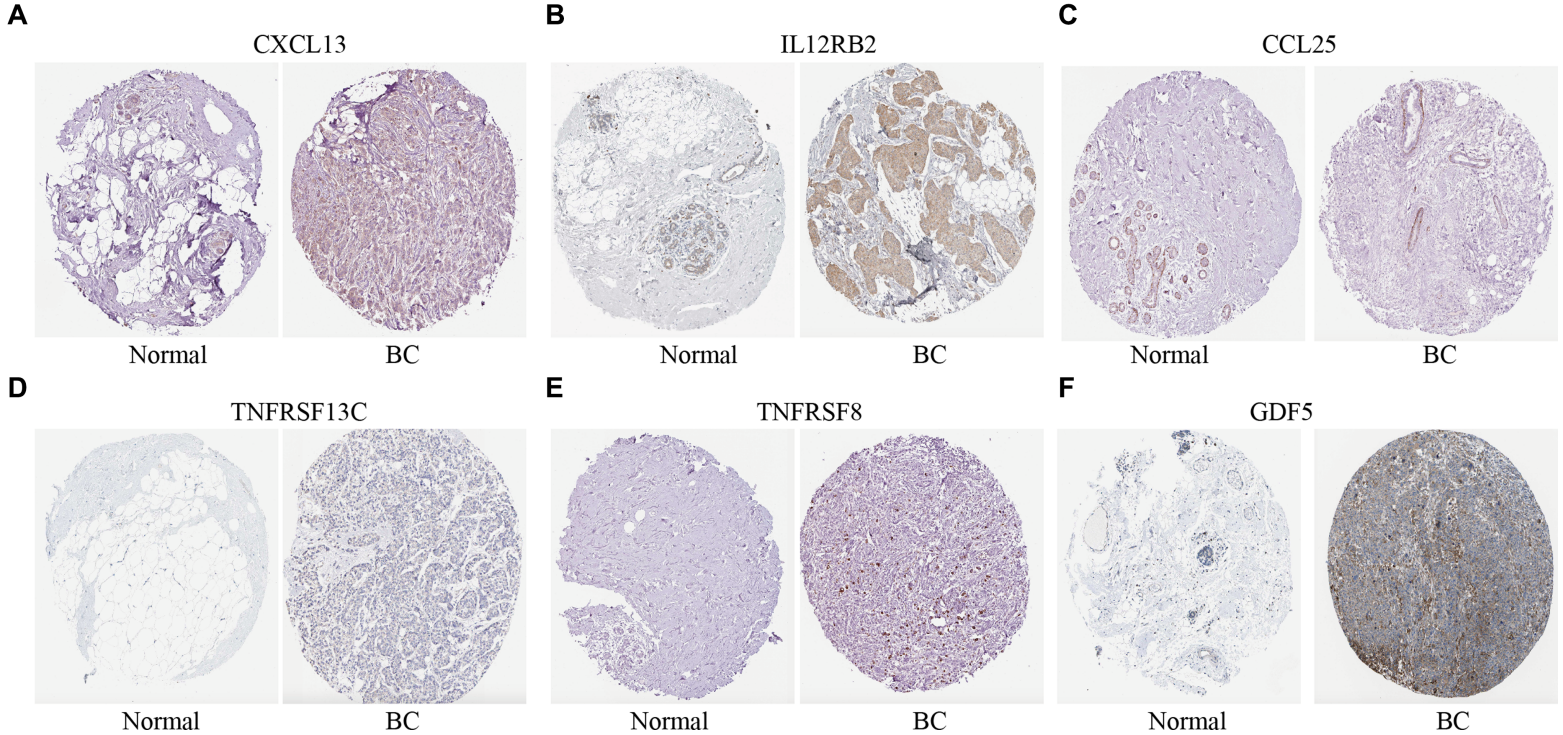
Immunohistochemical expression of the cytokine-related genes in BC tissues and normal tissues. **(A–F)** The immunohistochemical expression of six genes in normal tissues and BC tissues.

Immunohistochemical expression of eight genes were further validated between normal and BC tissues from the HPA database. Six genes (*CXCL13*, *IL12RB2*, *CCL25*, *TNFRSF13C*, *TNFRSF8*, and *GDF5*) were found in HPA database except *IL21* and *CCL7.* The expression of *CXCL13*, *IL12RB2*, *TNFRSF13C*, *TNFRSF8*, and *GDF5* in BC tissues were higher than normal tissues except *CCL25* (Figure 7).

## Discussion

In this study, we identified 499 prognostic genes in TNBC patients using data from TCGA database and identified the cytokine-related pathway closely related to TNBC based on functional enrichment analyses. Next, TCGA-TNBC patients were divided into the high-risk C1 and low-risk C2 groups based on the cytokine-related pathways by a consistency cluster analysis. The high-risk C1 patients exhibited tumor metastasis and an advanced tumor stage. The functional analysis revealed that the upregulated DEGs in C1 were mainly associated with the ECM-receptor interaction, focal adhesion, and the cAMP signaling pathway, while the downregulated DEGs in C1 were mainly associated with cytokine and cytokine receptors, Th17 cell differentiation, and primary immunodeficiency. We found that the immune activity of the high-risk C1 group was lower than that of the low-risk C2 group, and the IC50 scores of 3 chemotherapy drugs (i.e., doxorubicin, methotrexate, and paclitaxel) were lower in C2 group than C1 group. More importantly, we constructed a novel prognostic signature by a LASSO regression analysis and identified the following 8 genes: *CCL25*, *CXCL13*, *IL12RB2*, *IL21*, *TNFRSF13C*, *TNFRSF8*, *CCL7*, and *GDF5*. The results of the ROC and Kaplan–Meier curve analyses confirmed that the prognostic signature had good performance in predicting the prognosis of TNBC patients.

A systematic analysis revealed the cytokine-related pathway closely related to TNBC. The cytokines mainly included ILs, interferons, TNF superfamily, colony-stimulating factors, chemokines, and growth factors. Cytokines have pleiotropic effects in the TME. For example, IL-1 not only promotes inflammation, but also contributes to tumor invasion and angiogenesis. In addition, recombinant IL-2 has been approved by the FDA for the treatment of metastatic RCC and melanoma. Recombinant human IL-7 effectively induces an inflammatory TME. However, in BC, especially TNBC, the number of patients who will benefit from ICI treatment is small (14, 15). Thus, it is of great importance to identify novel biomarkers in TNBC.

The functional enrichment analysis of the C1 and C2 groups of the TNBC patients confirmed the cancer-related pathway (ECM-receptor interaction, stem cell proliferation, focal adhesion, and cAMP signaling pathway) and immune-related pathway (cytokine and cytokine receptors, Th17 cell differentiation, and primary immunodeficiency) related to TNBC. We further found that the immune activity of the high-risk C1 group was lower than that of the low-risk C2 group, and the IC50 scores of 3 chemotherapy drugs were lower in the low-risk C2 group. Thus, these results indicate that tumor classification could be used to distinguish TNBC based on the cytokine-related pathway.

Based on the strong correlation between the status of the cytokine-related pathway and the clinical prognosis of TNBC, we constructed a novel prognostic signature through a LASSO regression analysis and identified 8 genes (i.e., *CCL25*, *CXCL13*, *IL12RB2*, *IL21*, *TNFRSF13C*, *TNFRSF8*, *CCL7*, and *GDF5*) that predicted the prognosis of the TNBC patients well.

The thymus-expressed chemokine (*CCL25*) could enhance immunotherapy against TNBC by recruiting C-C chemokine receptor type 9 (CCR9) T cells (19). It has been reported that *CXCL13* produces an anti-tumor immune response in BC by regulating immune activity (20). *IL12RB2* and *TNFRSF8* are associated with the risk of lung adenocarcinoma (21). Moreover, IL-21 regulates proliferation, migration, and invasion in BC (22). *TNFRSF13C* could be a promising therapeutic target for TNBC (23). KLF15 suppresses tumor growth and metastasis in TNBC by *CCL7* (24). GDF5 regulates transforming growth factor beta-β-dependent angiogenesis in BC (25).

The present study had some limitations. The roles of the 8 genes should be further studied in TNBC. Moreover, the molecular mechanism should also be studied, which may provide new strategies for the treatment of TNBC.

In summary, the status of the cytokine-related pathway was closely related to tumor classification and immune activity in TNBC patients. Gene signatures of cytokine-related genes showed good performance in predicting the prognosis of TNBC patients. Our findings might provide new insights into the precision diagnosis and treatment of TNBC.

## Data availability statement

The datasets presented in this study can be found in online repositories. The names of the repository/repositories and accession number(s) can be found in the article/Supplementary material.

## Author contributions

XL and LC: conception and design and (V) data analysis and interpretation. LC: administrative support. XL, LZ, and LC: manuscript writing. All authors contributed to the article and approved the submitted version.

## Conflict of interest

The authors declare that the research was conducted in the absence of any commercial or financial relationships that could be construed as a potential conflict of interest.

## Publisher’s note

All claims expressed in this article are solely those of the authors and do not necessarily represent those of their affiliated organizations, or those of the publisher, the editors and the reviewers. Any product that may be evaluated in this article, or claim that may be made by its manufacturer, is not guaranteed or endorsed by the publisher.

## Footnotes

^1^ https://portal.gdc.cancer.gov/repository

^2^ https://www.cancerrxgene.org/

^3^ https://www.proteinatlas.org
